# Socioeconomic differentials in premature mortality in Rome: changes from 1990 to 2001

**DOI:** 10.1186/1471-2458-6-270

**Published:** 2006-11-02

**Authors:** Giulia Cesaroni, Nera Agabiti, Francesco Forastiere, Carla Ancona, Carlo A Perucci

**Affiliations:** 1 Department of Epidemiology, Rome E Health Authority, Via Santa Costanza 53, Rome 00198, Italy

## Abstract

**Background:**

While socioeconomic inequalities in mortality have widened in many countries, evidence of social differentials is scarce in Southern Europe. We studied temporal changes in premature mortality across socioeconomic groups in Rome between 1990 and 2001.

**Methods:**

We analysed all 126,511 death certificates of residents of Rome aged 0–74 years registered between 1990–2001. A 4-level census block index based on the 1991 census was used as an indicator of socioeconomic position (SEP). Using routine mortality data, standardised mortality rates (per 100,000 inhabitants) were calculated by SEP and gender for four time periods. Rate ratios were used to compare mortality by gender and age.

**Results:**

Overall premature mortality decreased in both genders and in all socioeconomic groups; the change was greater in the highest socio-economic group. In both men and women, inequalities in mortality strengthened during the 1990s and appeared to stabilise at the end of the 20th century. However, for 60–74 year old women the gap continued to widen.

**Conclusion:**

Socioeconomic inequalities in health in Rome are still present at the beginning of the 21^st ^century. Strategies to monitor the impact of SEP on mortality over time in different populations should be implemented to direct health policies.

## Background

Socioeconomic differences in mortality have been found all over Europe and in the US using individual and area-based socioeconomic indicators[1-3]. In several developed countries the gap in mortality between disadvantaged and well-off groups increased during the 1980s and the beginning of the 1990s[2,4-8]. The role of National Health Services (NHSs) and universal health care coverage in contrasting inequalities in health has been repeatedly advocated [9,10]. However, data on these disparities in Southern Europe, e.g. in Italy, in the second half of the 90s are rare[1,5,6]. In Rome, increases in mortality inequality have been documented in both genders for the periods 1990–92 and 1993–95[6]. In this study we evaluated the temporal changes of premature mortality (under 75 years of age) across socioeconomic groups in Rome over a period of 12 years, from 1990 to 2001.

## Methods

Rome (2,800,000 inhabitants) is divided into 5736 census blocks with an average of 480 inhabitants in each. The area-based socioeconomic position indicator (SEP) was derived from the 1991 census and has been described elsewhere[6]. Briefly, we considered educational level, occupation, household condition, and number of residents per house per block, and divided the blocks into four categories, from very well off (level I) to very underprivileged (level IV). We used mortality data from the Regional Registry of Causes of Death. Individual records include demographic data, underlying cause of death, and census block of residence (CB), but do not contain individual information on socioeconomic position, such as level of education, income, or occupation. Between 1990–2001, 126.511 premature deaths for Roman residents (regardless of city of death) were recorded. We excluded 5.206 records because the census block of residence was not reported (4.1% of deaths). We analysed all-cause mortality for people aged 0–74 years, and three age categories in particular: 20–44 years, 45–59 years, and 60–74 years. We obtained from the Municipal Registry Office of Rome the census block populations for 1991, 1995, 1999, and 2000, divided into 16 age groups. The values of the remaining years were calculated by linear interpolation. We computed mortality rates by SEP level for four time periods: 1990–92, 1993–95, 1996–98, and 1999–2001. Rates (per 100,000 inhabitants) were stratified by gender and standardised by age (direct method, European population as the standard, 5-year age groups). We used rate ratios (RRs) with 95% confidence intervals (95%CI) to compare inequalities in mortality by gender and age.

## Results

Table 1 shows mortality rates of the most affluent group and RRs of most underprivileged vs. most affluent in four 3-year periods. Both men and women in the lowest socioeconomic group had a greater risk of dying than those in the highest group. This effect was remarkably greater for men than for women, and for those aged 20–44. Overall mortality decreased in the study period in both genders and in all socioeconomic groups (1999–2001 vs. 1990–1992 in men and women: -30% and -19% most affluent group, -18% and -13% most underprivileged group). The rate ratios increased from 1990–1992 (1.24 for men and 1.15 for women) to 1996–1998 (1.50 for men and 1.28 for women) and then levelled off in both genders in the last study period. Inequalities emerged during the second half of the study for women aged 45–59 and continuously increased in those aged 60–74 year. In young adults, the highest RRs were found in 1996–98 in both genders, but they decreased slightly in the last study period.

## Discussion

Overall mortality decreased in both genders, especially for those living in the most affluent CBs. The increasing mortality differential by SEP in the 1990s stabilized at the beginning of the new millennium, except in women over 44 years of age for whom the gap continued to widen, and in young adults for whom the gap decreased.

Our study confirms the general evidence that socioeconomic differences in mortality for all causes increased in the first half of 1990s mainly because of faster proportional declines in death rates among those of high SEP, with stronger effects among young men[4,7,11]. Previous studies examined specific causes of death and highlighted possible mechanisms of the increase in mortality inequalities by gender and age-category, and of their variation across countries[1,4,8,12]. In Rome and in other European cities, AIDS and drug abuse emerged as responsible for the widening gap, especially among men, while in Finland increasing death rates in the manual labour class for alcohol-related causes, accidents, and suicide were the main causes[5,6,13,14]. Based on a recent international comparison across European countries among middle-aged people, the increase in disparities was mainly due to a faster decline in cardiovascular disease mortality in higher socioeconomic groups and to increasing mortality rates in the lower socioeconomic group for other causes (lung cancer, breast cancer, respiratory diseases, gastrointestinal disease, and injuries)[4]. However, as "an exception in Europe", Italy (Turin) showed a faster decline of cardiovascular mortality in the lower socioeconomic groups[4].

Changes in health-related behaviours have been proposed as one of the main determinants of differences between populations. Among them, smoking is an important risk factor for disease and its socioeconomic inequalities strongly vary by age group[15,16]. In the last decades, smoking had a faster decline in the upper than in the lower SEP groups in Northern Europe. This phenomenon might have partially influenced the widening socioeconomic gradient in cardiovascular disease in countries like England/Wales, Finland, Denmark, Sweden and Norway in the 1990s[4,17]. Differences in diet, prevalence of obesity and other cardiovascular risk factors are other possible candidates[18]. Lastly, the more pronounced SEP differential for cerebrovascular disease and cardiovascular disease other than ischemic (i.e. heart failure) in women and in the elderly suggests socioeconomic differences in disease detection and treatment as possible explanations[17]. It should be considered, however, that behavioural factors are only a part of the explanation of socioeconomic disparities since they act as "proximal risk factors" in the complex conceptual model of health inequalities more influenced by "distal social determinants" like income-inequality environment, employment status and job security, and health care[19]. The time lags between exposure to different risk factors and their potential effects on specific health outcomes play an important role and should be taken into account while interpreting temporal patterns of mortality[20].

Health is strongly influenced by social determinants, which play a role throughout life, e.g. childhood circumstances, employment opportunities and environment, household living conditions, access, knowledge, and utilization of high quality health care services. Therefore, changes over time in structural and economic characteristics of societies are other potential determinants of the widening differential in mortality; however, the mechanisms are complex[21-23]. In particular, the extent to which health care influences health inequalities has not completely elucidated[24,25]. In the Netherlands it has been suggested that both higher and lower SEP groups may have benefited from mortality reductions in the last decades because of largely equal access to essential health services, however there is no evidence that health care utilization has influenced the widening inequalities in health[24]. Inequities in access to procedures such as bypass and angiography have been reported in Europe, but the extent to which health care disparities contribute to socioeconomic disparities in overall mortality is still uncertain[26-28].

New changes in overall mortality at the end of the millennium have been studied only in Britain: they observed an increase in the relative index of inequality for mortality until 1999, which paralleled trends in income inequalities, particularly in younger men [7]. The reduced gap between socioeconomic groups among the youngest adults in Rome in 1999–2001 presents an interesting contrast. One possible explanation might be the decline in AIDS mortality since 1997. Increasingly diffuse HIV treatments have helped to equalise the length of AIDS survival across socioeconomic groups[29,30]. The spread of HIV incidence from drug users to the heterosexual population might also reduce the impact of the AIDS epidemic on more deprived populations[31]. Other possible explanations include the slight decline over time in drug overdose mortality and the decrease in serious brain injuries after the introduction of the Italian motorcycle helmet law in January 2000[32,33]. A general downward trend in smoking prevalence from 1950 to 2000 has been noted in Italy, with increased inequalities for young men and women over time[34,35]. On the other hand, in an international comparison across European countries, the authors suggest that the efforts adopted since the 1980s in Italy (pricing policy, ban of promotion of tobacco products, restriction of smoking in indoor places) may have been more effective among low SEP men to avoid smoking initiation and encourage cessation[15]. However it is difficult to believe that such changes are responsible for the reduced disparities in mortality among 25–44 years aged adults in the last period of our study.

Validity aspects of the study should be mentioned. Because of the lack of individual information in routine mortality data, we used a small area index rather than an individual measure of socioeconomic position. In evaluating the results, it should be underlined that we are dealing with changes of area-level socioeconomic inequalities not of individual SEP measures. However, small area indexes can be considered *per se *a valid measure of SEP: areas of great underprivilege may also be disadvantaged with respect to social organisation, transportation, pollution, healthcare facilities, and other factors that might influence health. In the last 15 years geographical indicators have been used in many developed countries, usually derived by census or administrative data. The United Kingdom is the European country with the strongest tradition in use of small area SEP indices for public health purposes[36-38]. In the US an important contribution was made by the 'The Public Health Disparities Geocoding Project', aimed at monitoring US socioeconomic inequalities in health and at understanding which indices can be used, and at which level to succeed in the objective[39-41]. Population denominators were available for four years only, so we used linear interpolation for the remaining years. The exclusion of 4.1% of deaths because of missing census block information is not likely to have biased the results. In fact, there is no reason to think that the missing census information is associated in any way to the area of residence, or to SEP. To measure the association between SEP and mortality we used rate ratios of extreme SEP index levels instead of a relative index of inequality[42]. However, there was no significant change in the distribution of the population across levels of the indicator over time.

As a final remark, the strong social, economic, behavioural and environmental forces that drive most of the inequalities in health in our society cannot be effectively contrasted by health care interventions alone. Social and welfare policies that play an important part in determining health, but are outside the immediate control of the health sector, are clearly needed. The choice of such comprehensive equitable social policies, as well as the decisions regarding "acceptable" and "non-acceptable" levels of health inequality, are the result of political decisions and legislation driven by societal ethical values of distributive justice[43,44]. Unfortunately no national policies on health inequalities have been set in Italy, which would provide a clear benchmark for a longitudinal evaluation.

## Conclusion

There are still substantial socioeconomic inequalities in health in Rome, and the magnitude of the association is similar to that found in other Western countries. Since factors involved in growing social inequalities may be different across countries, it is essential that each country develops a specific programme to monitor population health for different SEP groups and to tackle disparities[45]. From a public health perspective it is then essential to target lower SEP groups to reduce prevalence of risk factors, and to facilitate access to the best available health services.

## Abbreviations

SEP socioeconomic position

NHS National Health Services

RR rate ratios

AIDS Acquired Immunodeficiency Syndrome

HIV human immunodeficiency virus

## Competing interests

The author(s) declare that they have no competing interests.

## Authors' contributions

All authors participated in the design of the study, the definition of the statistical analyses, and in the discussion of the results. All authors read and approved the final manuscript. GC conceived the study, performed the statistical analysis, and drafted the manuscript. NA conceived the study and drafted the manuscript.

## Pre-publication history

The pre-publication history for this paper can be accessed here:



## Figures and Tables

**Table 1:** All-cause mortality in Rome from 1990 to 2001 by socioeconomic position (age 0–74 years).

	**Men**															
	**1990–92**				**1993–95**				**1996–98**				**1999–2001**			
	**deaths**	**rate**	**RR**	**95%CI**	**deaths**	**rate**	**RR**	**95%CI**	**deaths**	**rate**	**RR**	**95%CI**	**deaths**	**Rate**	**RR**	**95%CI**
	3528	455	**1.24**	**1.19 – 1.30**	3255	402	**1.35**	**1.29 – 1.41**	2772	335	**1.50**	**1.43 – 1.58**	2582	318	**1.46**	**1.39 – 1.54**
**Age (years)**																
**20–44**	312	120	**1.62**	**1.41 – 1.86**	297	120	**1.78**	**1.55 – 2.04**	237	96	**1.87**	**1.61 – 2.17**	207	84	**1.66**	**1.41 – 1.96**
**45–59**	675	497	**1.23**	**1.11 – 1.35**	618	444	**1.34**	**1.21 – 1.48**	505	360	**1.54**	**1.38 – 1.72**	502	359	**1.51**	**1.40 – 1.68**
**60–74**	4960	2328	**1.18**	**1.11 – 1.25**	4576	2010	**1.30**	**1.22 – 1.37**	3986	1718	**1.42**	**1.35 – 1.51**	3676	1618	**1.40**	**1.32 – 1.49**

	**Women**															
	**1990–92**				**1993–95**				**1996–98**				**1999–2001**			
	**deaths**	**rate**	**RR**	**95%CI**	**deaths**	**rate**	**RR**	**95%CI**	**deaths**	**rate**	**RR**	**95%CI**	**deaths**	**Rate**	**RR**	**95%CI**

	2387	224	**1.15**	**1.09 – 1.23**	2352	218	**1.14**	**1.07 – 1.21**	2071	195	**1.28**	**1.20 – 1.36**	1853	182	**1.23**	**1.15 – 1.31**
**Age (years)**																
**20–44**	163	61	**1.34**	**1.10 – 1.63**	165	63	**1.29**	**1.06 – 1.58**	134	50	**1.62**	**1.32 – 2.00**	123	45	**1.34**	**1.07 – 1.67**
**45–59**	460	283	1.01	0.89 – 1.15	412	255	1.11	0.97 – 1.26	412	251	**1.14**	**1.00 – 1.30**	356	218	**1.19**	**1.04 – 1.37**
**60–74**	1728	1058	**1.16**	**1.08 – 1.30**	1734	1041	**1.15**	**1.07 – 1.23**	1496	928	**1.27**	**1.18 – 1.40**	1327	845	**1.30**	**1.21 – 1.40**

Legend: Number of deaths and mortality rate (x100,000) in level I (well off), and rate ratios in level IV (underprivileged) to that in level I
